# One‐Step Coaxial 3D Printing of Pre‐Vascularized Skin Organoid Models with ADSC Microspheres for Enhanced Wound Healing

**DOI:** 10.1002/advs.202517409

**Published:** 2025-11-29

**Authors:** Kunrui Wang, Xiang Lan, Jianwei Chen, Yu Wu, Delong Zhu, Xiangkai Kong, Ying Hu, Qian Liu, Kun Wang, Tao Xu, Lei Zhu

**Affiliations:** ^1^ Third Affiliated Hospital of Sun Yat‐Sen University Department of Dermatology and Plastic Surgery Sun Yat‐Sen University No.600 Tianhe Road, Tianhe Guangzhou Guangdong 510630 China; ^2^ Center for Bio‐Intelligent Manufacturing and Living Matter Bioprinting Research Institute of Tsinghua University in Shenzhen Tsinghua University Shenzhen 518000 China; ^3^ Department of Research and Development Qingyuan Zhixin (Shenzhen) Biotechnology Co., Ltd Shenzhen Guangdong 518000 China; ^4^ 3D Printing Bio‐Intelligent Manufacturing and Application Engineering Research Center Of Guangxizhuang Autonomous Region Nanning 530021 China; ^5^ Third Affiliated Hospital of Sun Yat‐Sen University Department of Orthopedic Surgery Sun Yat‐Sen University No.600 Tianhe Road, Tianhe Guangzhou Guangdong 510630 China; ^6^ Department of Ophthalmology Ninth People's Hospital Shanghai JiaoTong University School of Medicine Shanghai 200011 China

**Keywords:** 3d printing, adipose‐derived stem cells, microspheres, pre‐vascularized, skin organoid, vascular bed, wound healing

## Abstract

Organoids are important tools for studying organ development, drug screening, and regenerative medicine, yet the absence of integrated vasculature limits their culture and translation. To address this, the PV‐XOM strategy is proposed, which achieves one‐step construction of pre‐vascularized organoids through coaxial bioprinting: the inner phase uses temperature‐responsive sacrificial material and endothelial cells to form hollow vascular channels, while the outer phase is a biomimetic hydrogel matrix containing organoid microspheres. Based on this framework, a pre‐vascularized skin organoid model (PV‐SOM) is established, in which the outer phase is loaded with adipose‐derived stem cell (ADSC) microspheres and skin fibroblasts. In vitro, PV‐SOM achieved rapid vascular closure and maturation; in vivo, it formed abundant neovessels in large skin defects, accelerated wound closure, and improved collagen remodeling. Proteomic analysis further revealed that ADSC microspheres activate the PI3K–AKT–mTOR pathway to regulate vascular formation across multiple stages. These findings show that PV‐XOM offers an effective, scalable solution to the vascularization bottleneck of organoids with strong translational potential.

## Graphical abstract

A multifunctional vascular bed platform is created using coaxial 3D printing, allowing the one‐step integration of organoid microspheres with perfusable vascular structures. This strategy enables rapid and functional vascularization of organoids to support tissue repair and vascularization, providing a scalable and translationally promising approach for advancing organ regeneration therapies.

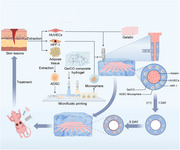

## 1 Introduction

Organoids derived from pluripotent stem cells, embryonic stem cells, or adult stem cells represent 3D culture systems that can recapitulate the complex architecture and functions of native organs.^[^ ^1^ ^]^ With advances in fabrication technologies, organoids have become powerful tools for studying organ development, disease modeling, and drug screening.^[^ ^2^ ^]^ However, the absence of vascular networks remains a critical bottleneck limiting their translational potential and the ability to mimic complex in vivo functions.^[^ ^1^, ^2^ ^]^ Vascularization is indispensable for organoids, as it mediates nutrient and waste exchange and determines long‐term stability and functional maintenance during culture.^[^ ^2^, ^3^, ^4^, ^5^, ^6^, ^7^ ^]^

The emergence of vascular organoids has provided new insights into addressing this challenge. Wimmer et al. (2019) generated vascular organoids from iPSCs that formed stable, perfusable vascular trees after transplantation into mice,^[^ ^6^ ^]^ while Marina et al. (2022) mapped the developmental trajectory of iPSC‐derived vascular organoids.^[^ ^7^ ^]^ Although these studies advanced vascular disease modeling, their structures were confined to isolated vascular networks and were difficult to integrate with target organoids.

Despite their ability to self‐organize and partially mimic native tissue architecture, conventional stem‐cell–derived organoids still face major limitations, including insufficient maturation, lack of mechanical stability, and the absence of vascular support. Recent studies have emphasized that engineering‐assisted organoid platforms can effectively overcome these constraints. Jin et al.^[^ ^8^ ^]^ demonstrated that introducing defined microenvironmental cues and biomaterial scaffolds markedly improves organoid reproducibility and functional performance across multiple tissues. Similarly, Shao et al.^[^ ^9^ ^]^ highlighted that ECM‐mimicking hydrogels and bioactive matrices enhance structural stability and promote lineage‐specific differentiation. Moreover, Hsiung et al.^[^ ^10^ ^]^proposed integrating organoids with tissue‐engineering tools—such as 3D bioprinting and microfluidics—to achieve more precise architectural control and support vascular incorporation. Collectively, these advances indicate that engineered organoid‐based constructs, which couple self‐organization with structural guidance, offer a promising direction for developing more mature and translationally relevant models. This framework provides strong justification for our development of a pre‐vascularized skin organoid model (PV‐SOM).

Current strategies for organoid vascularization fall into three main categories: pre‐patterning lumens using sacrificial templates followed by endothelial cell perfusion, co‐culture with endothelial cells, or co‐culture with vascular organoids.^[^ ^2^ ^]^ For example, Ouyang et al. (2019) created seamless vascular chips using temperature‐responsive sacrificial materials;^[^ ^11^ ^]^ Zhang et al. (2025) enhanced vascularization in skin organoids by incorporating endothelial cells;^[^ ^12^ ^]^ and Ahn et al. (2021) introduced endothelial cells into brain organoids on day 5 of culture and observed vascular ingrowth in vivo.^[^ ^4^ ^]^ However, these methods often suffer from disorganized vascular networks, low perfusion efficiency, technical complexity, and poor reproducibility. Thus, a one‐step approach to constructing integrated vascularized organoids remains an urgent challenge.^[^ ^1^, ^4^ ^]^ Coaxial 3D printing, with its capacity for simultaneous extrusion of inner and outer phases, enables the fabrication of perfusable lumens in the inner phase while synchronously embedding organoid cells in the outer phase^[^ ^13^, ^14^, ^15^ ^]^ (Figure S1, Supporting Information). This offers a promising route toward one‐step formation of integrated vascularized organoids.

For vascular structures to achieve long‐term maturation and stability, additional biological signaling beyond hollow channels is essential.^[^ ^16^ ^]^ Previous studies have often relied on growth factors such as VEGF to promote angiogenesis,^[^ ^17^, ^18^, ^19^ ^]^ but their transient release and limited signaling profiles are insufficient to sustain vascular development. Adipose‐derived mesenchymal stem cells (ADSCs), by contrast, possess strong paracrine activity and continuously secrete a wide spectrum of cytokines covering multiple stages of angiogenesis,^[^ ^20^ ^]^ making them an ideal “cellular drug” to promote vascular maturation.^[^ ^19^, ^21^, ^22^ ^]^ Microfluidic technology enables scalable and uniform production of ADSC microspheres, which maintain 3D self‐assembly, strengthen cell–cell and cell–matrix interactions, and enhance paracrine efficiency.^[^ ^14^, ^23^ ^]^ These microspheres act as “cellular pharmacies” for sustained signal release and can be used as bio‐inks for 3D printing. Importantly, microfluidics provides a stable microenvironment that protects cells from shear stress during extrusion.^[^ ^23^, ^24^ ^]^

The extracellular matrix (ECM) is also critical for organoid construction. Commonly used hydrogels, such as HAMA or GelMA, exhibit printability, but HAMA lacks cell‐adhesion sites and GelMA, derived from gelatin fragments, lacks the native collagen triple‐helix structure, limiting biomimicry.^[^ ^19^, ^25^ ^]^ Materials based on type I collagen have drawn attention due to their close resemblance to natural ECM. Methacrylated type I collagen (Col1MA) retains the triple‐helix structure and offers excellent cell adhesiveness and tissue integration.^[^ ^26^, ^27^ ^]^ Combining Col1MA with GelMA ensures printability while providing a highly biomimetic ECM environment, thereby strongly supporting both organoid development and vascularization.

Based on these principles, we propose the PV‐XOM (Pre‐Vascularized X‐Organoid Model) strategy, where “X” denotes the target tissue type, enabling a modular and generalizable approach to constructing vascularized organoids. This strategy employs coaxial printing to achieve one‐step construction of integrated perfusable vascularized organoids: the inner phase comprises temperature‐responsive sacrificial gelatin and endothelial cells, which form hollow lumens after printing, while the outer phase consists of organoid microspheres generated via microfluidics embedded in a Col1MA–GelMA biomimetic matrix.

To showcase its translational potential, we applied PV‐XOM to skin organoid engineering. Currently, skin organoids lack effective vascularization strategies, while clinically used artificial skin grafts for large‐area wound repair are limited by insufficient vascularization and poor host integration.^[^ ^12^, ^28^, ^29^ ^]^ Against this backdrop, we constructed a pre‐vascularized skin organoid model (PV‐SOM) by integrating ADSC microspheres, dermal fibroblasts, human epidermal keratinocytes, and pre‐vascularized networks. We systematically evaluated its mechanical and biomimetic material properties, the construction and maturation of integrated vascular networks, and the paracrine role of ADSC microspheres in promoting angiogenesis and cell proliferation. Furthermore, transplantation experiments demonstrated that PV‐SOM markedly enhanced vascularization and accelerated the repair of large skin defects in vivo.

**Figure 1:**
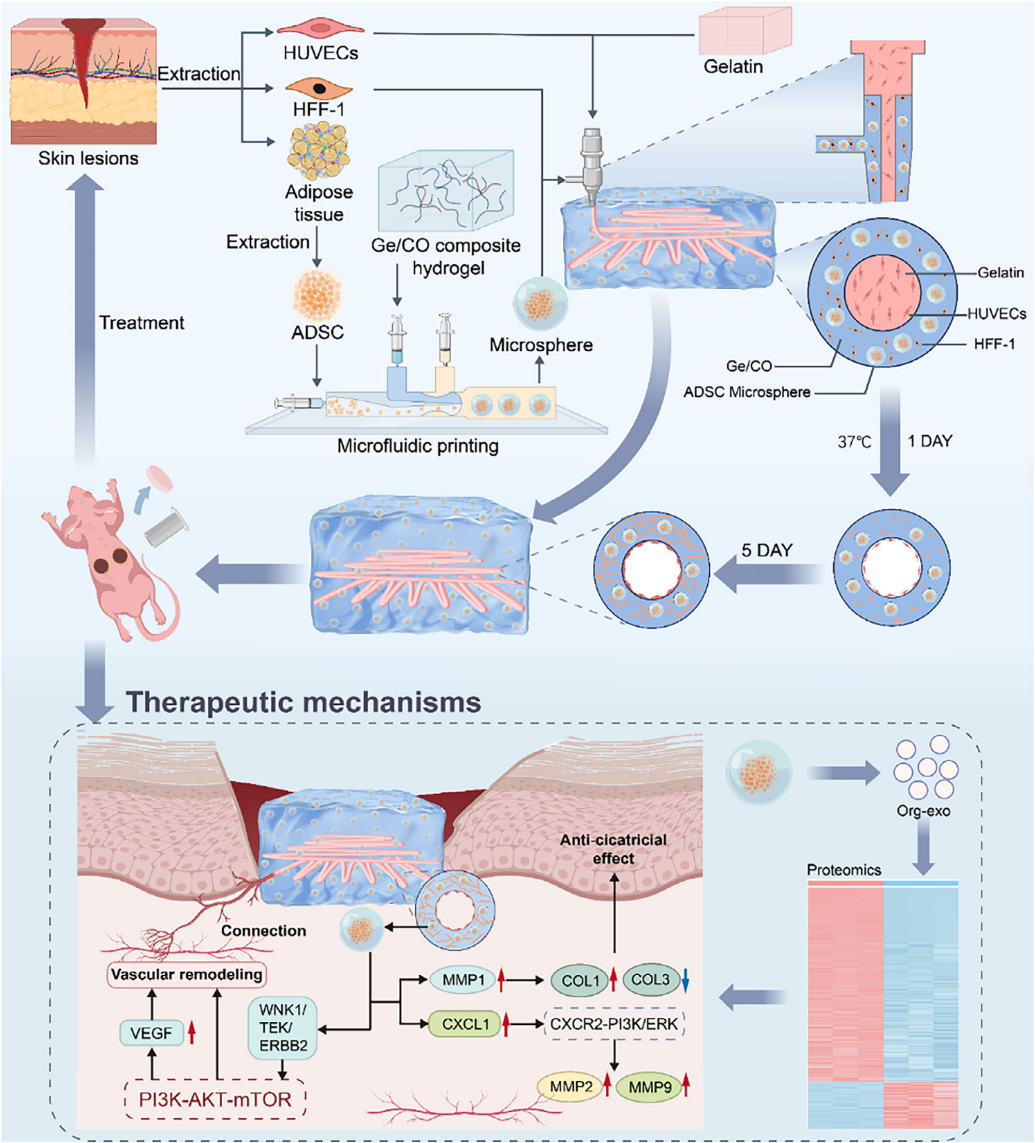
Schematic illustration of the development of a pre‐vascularized skin organoid model (PV‐SOM) based on PV‐XOM. This strategy integrates HFF‐1 cells with ADSC microspheres in a one‐step process on a vascular bed. The upper panel depicts the design and fabrication of the organoid model, in which microfluidics enables high‐throughput preparation of self‐assembled ADSC microspheres, while coaxial 3D printing constructs perfusable vascular channels within a GE/CO hydrogel matrix loaded with cells and microspheres. The lower panel illustrates the application of PV‐SOM in treating full‐thickness skin defects in mice and its underlying mechanisms of action.

In conclusion, by integrating coaxial 3D printing with microfluidic technology, PV‐XOM provides a versatile and scalable strategy to overcome the vascularization bottleneck in organoids. This platform not only enables modular and uniform construction of pre‐vascularized organoids but also holds strong clinical translational potential in regenerative medicine (**Figure** **1**).

## 2 Results

### 2.1 Characterization of GelMA–Col1MA Composite Hydrogels

GelMA hydrogels, owing to their excellent biocompatibility and tunable physical properties, have been widely applied in tissue regeneration, drug delivery, and tissue adhesion. Building upon this foundation, we introduced methacrylated type I collagen (Col1MA) and blended it with GelMA to fabricate a composite hydrogel (GE/CO).

**Figure 2:**
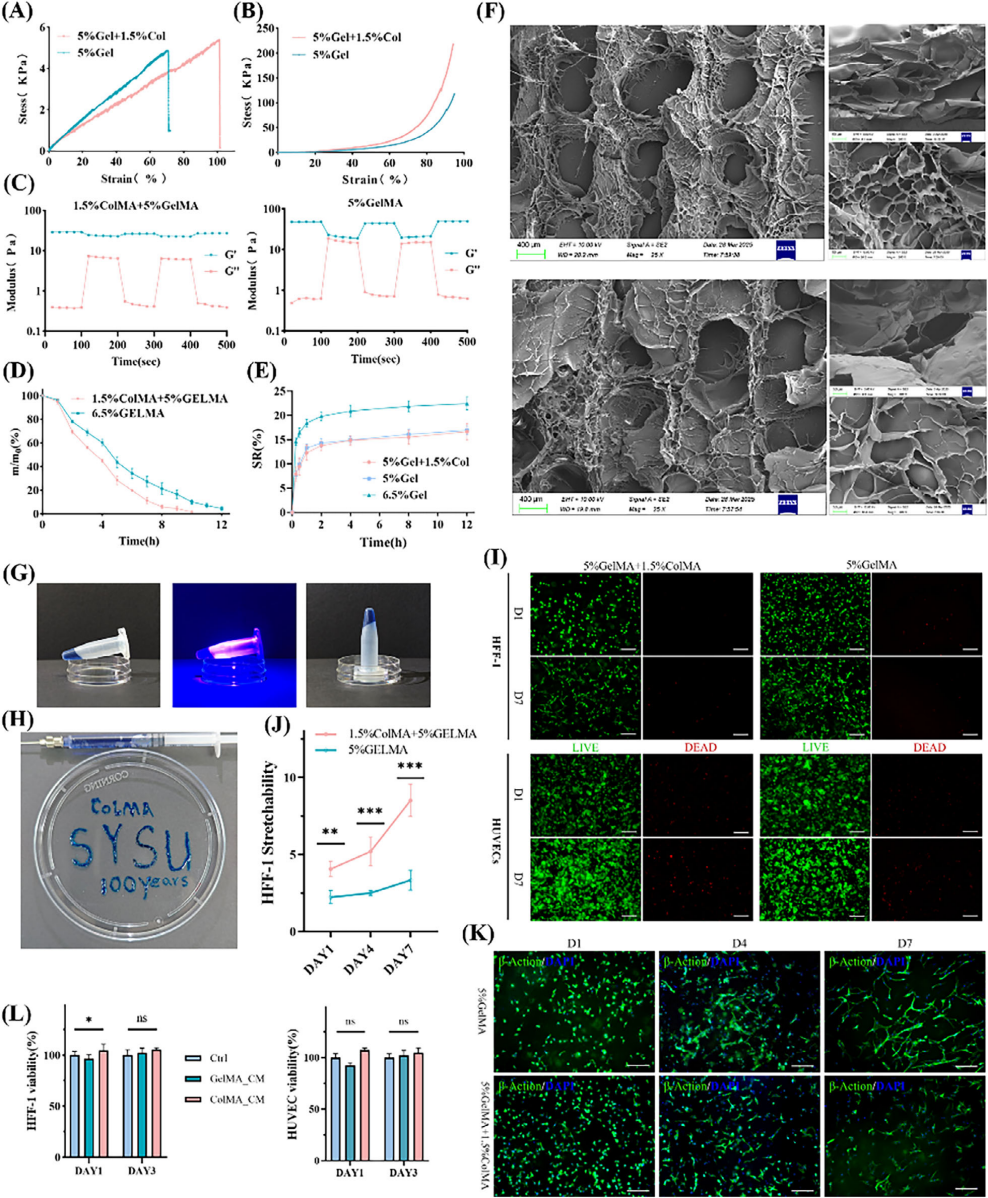
Physicochemical characterization and biocompatibility assessment of GE/CO hydrogels. including A) tensile stress–strain curves of GE/CO and GelMA hydrogels, B) compressive stress–strain curves, C) rheological analysis showing shear‐thinning behavior, D) collagenase degradation assay, E) swelling ratio measurements, F) high‐resolution scanning electron microscopy (HR‐SEM) images of GE/CO (top) and GelMA (bottom) hydrogels (scale bar: 400 µm), G) photographs of hydrogels after 405 nm blue‐light crosslinking, H) printability test, I) Live/Dead staining images of HFF‐1 and HUVECs cultured on hydrogels at days 1 and 7 (scale bar: 200 µm), J,K) cytoskeleton staining images of HFF‐1 cells cultured at days 1, 4, and 7 with corresponding quantitative analysis (*n* = 4, mean ± SD; Non‐paired *t*‐test; scale bar: 200 µm), and L) CCK‐8 assay. (*n* = 3, mean ± SD; One‐way ANOVA followed by Tukey's multiple comparisons). With statistical significance indicated as ^*^ *p* < 0.05, ^**^ *p* < 0.01, ^***^ *p* < 0.001, ns non‐significant.

Mechanical testing revealed that GE/CO exhibited significantly higher tensile strength and elongation at break compared with pure GelMA (**Figure** **2**A), and also demonstrated greater resistance to deformation under compression (Figure 2B). Rheological analysis showed that both hydrogels displayed shear‐thinning behavior (Figure 2C); however, GE/CO presented a markedly higher storage modulus (*G*′) across the entire frequency range.

In vitro degradation assays indicated that GE/CO degraded slightly faster than pure GelMA in the presence of 0.1% collagenase (Figure 2D). Swelling experiments revealed that the swelling ratio of GE/CO was significantly lower than that of 6.5% GelMA (reduced from 22.4% to 16.6%), approaching the level of 5% GelMA (Figure 2E).

Scanning electron microscopy demonstrated that GE/CO possessed a uniform, dense porous structure with fibrous surface textures (Figure 2F), whereas pure GelMA exhibited a looser structure with poorly supported pore walls. Photocrosslinking tests confirmed that GE/CO could rapidly form gels within 20 s under blue light irradiation (Figure 2G), and subsequent printing tests further verified its excellent line fidelity and structural precision (Figure 2H).

In cell compatibility assays, Live/Dead staining showed that GE/CO maintained high viability of HUVECs and HFF‐1 cells on both day 1 and day 7 of culture (Figure 2I). F‐actin staining revealed that HFF‐1 cells within GE/CO displayed well‐spread morphology and interconnected networks, whereas cells in pure GelMA appeared rounded with limited spreading. Quantitative analysis confirmed a significant increase in HFF‐1 spreading area in GE/CO at days 4 and 7 (*p* < 0.001) (Figure 2J,K). Moreover, CCK‐8 assays indicated that both GE/CO and pure GelMA showed no significant difference in cell viability compared with the control group (Figure 2L).

### 2.2 Fabrication and Characterization of Vascular Beds via Coaxial Bioprinting

**Figure 3:**
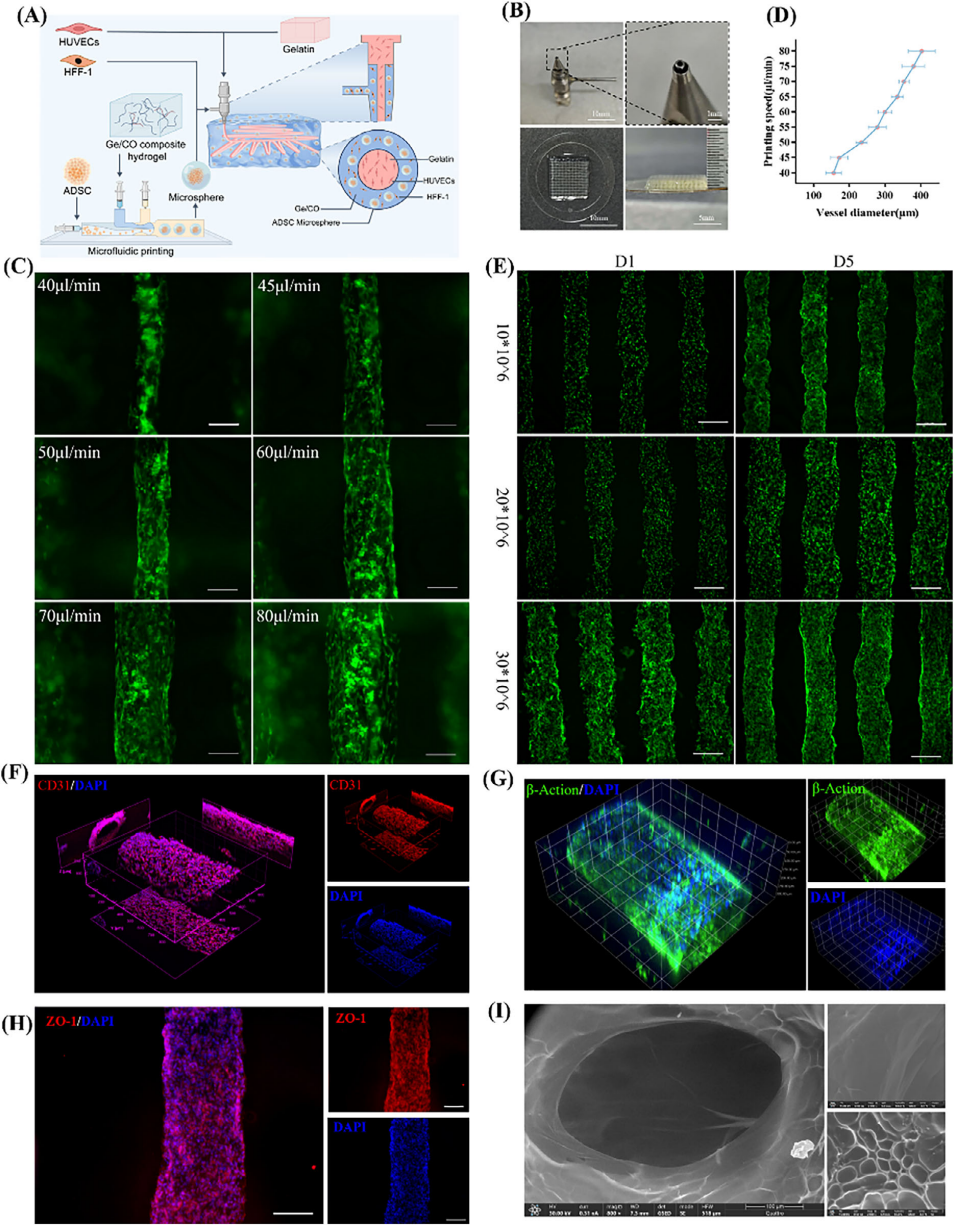
Fabrication and Characterization of Vascular Beds via Coaxial Bioprinting. including A) schematic illustration of coaxial 3D bioprinting, B) photographs of the printing nozzle and printed constructs, C) fluorescent images of hollow channels formed under different core flow rates with GFP‐labeled HUVECs (green) (scale bar: 200 µm), D) quantitative analysis of lumen diameters corresponding to (C) (*n* = 5, mean ± SD), E) fluorescent images of channels formed with different HUVEC densities in the core phase (scale bar: 200 µm), F) immunofluorescence staining of CD31 at day 5, G) fluorescent staining of F‐actin (green) and DAPI (blue) at day 5, H) immunostaining of ZO‐1 at day 5 (scale bar: 200 µm), and I) environmental scanning electron microscopy (ESEM) images.

The fabrication principle of the pre‐vascularized skin organoid model is illustrated in **Figure** **3**A. The shell layer consists of a GE/CO hydrogel encapsulating fibroblasts and ADSC microspheres, while the core layer is composed of a gelatin solution containing HUVECs. The actual coaxial nozzle structure and printed constructs are shown in Figure 3B, demonstrating the alignment and coordinated extrusion of the inner and outer phase fluids.

With respect to vascular channel diameter regulation, increasing the core flow rate proportionally enlarged the lumen diameter (Figure 3C), and quantitative analysis confirmed flexible control within a range of ≈80–250 µm (Figure 3D). Endothelial cell density also affected lumen formation: at high density, cells established compact intercellular connections and continuous coverage, whereas low density resulted in incomplete alignment and loose edges (Figure 3E).

Immunofluorescence staining demonstrated a continuous circular distribution of CD31 in HUVECs at day 5 (Figure 3F), indicating the formation of a confluent endothelial lining. F‐actin staining revealed circumferentially arranged actin filaments (Figure 3G), consistent with cytoskeletal remodeling during vascular development. ZO‐1 staining confirmed the establishment of continuous tight junction belts between adjacent cells (Figure 3H). Environmental scanning electron microscopy (ESEM) further revealed continuous lumens with smooth inner walls (Figure 3I). Meanwhile, we observed that the inner axis cells flowed within the lumen after printing (Figure S3, Supporting Information).

To investigate whether the endothelial cells in the scaffold were in direct contact and tightly connected within the hollow channels, we used GelMA lysis buffer to dissolve the supporting material GelMA in the PV‐SOM on day 7. The GelMA lysis buffer completely dissolved the GelMA within minutes without disrupting the cell connections. In the dissolution results, we can see that after scaffold dissolution on day 7, GFP‐HUVECs were interconnected in a cord‐like structure rather than as scattered individual cells (Figure S2, Supporting Information).

### 2.3 Microfluidic Printing of ADSC Microspheres and Their Functional Standards and Mechanisms

**Figure 4:**
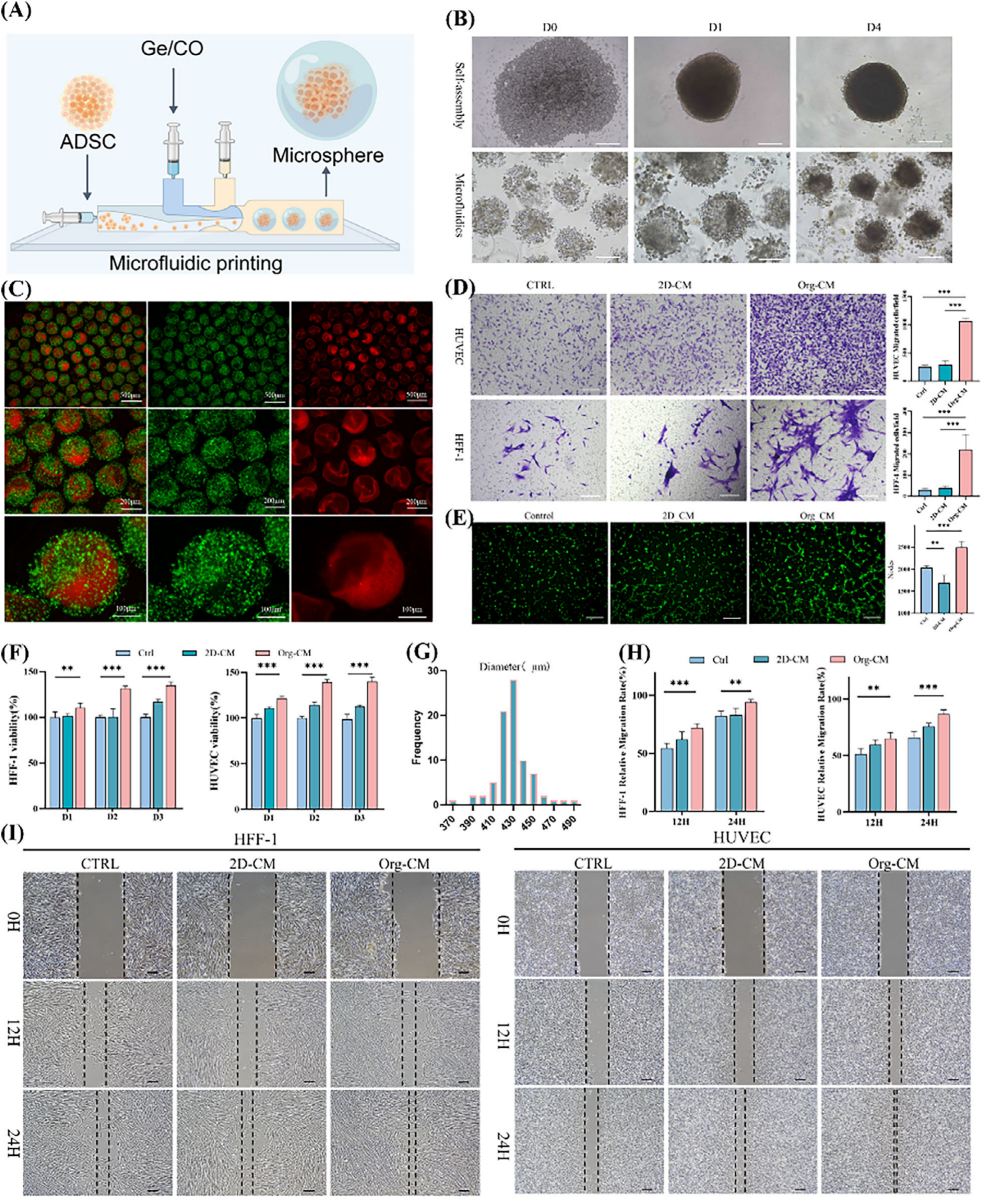
Characterization and functional assessment of ADSC microspheres fabricated by microfluidics. A) Schematic illustration of microfluidic fabrication of core–shell ADSC microspheres; B) comparison of ADSC self‐assembly within microspheres and in ultra‐low attachment plates; C) fluorescent labeling with red and green nanoparticles in core and shell layers to verify spatial organization; D) Transwell Migration Assay (*n* = 5, mean ± SD; One‐way ANOVA followed by Tukey's multiple comparisons); E) tube formation assay of HUVECs with quantification of branch points(*n* = 5, mean ± SD; One‐way ANOVA followed by Tukey's multiple comparisons.); F) CCK‐8 assay of cell proliferation(*n* = 5, mean ± SD; One‐way ANOVA followed by Tukey's multiple comparisons); G) size distribution analysis of microspheres (*n* = 100); h,i) scratch wound assay and quantitative analysis. Statistical significance is indicated as ^*^ *p* < 0.05, ^**^ *p* < 0.01, ^***^ *p* < 0.001, ns non‐significant.

To enhance the maturation and stability of pre‐vascular structures while strengthening wound repair capacity, adipose‐derived mesenchymal stem cells (ADSCs) were introduced as paracrine “cellular drugs.” Using a microfluidic approach, core–shell structured ADSC microspheres were fabricated (**Figure** **4**A). The core–shell dual‐channel microfluidic printing chip, organoid printer, and printing process are shown in Figure S4 (Supporting Information). Comparative experiments demonstrated that ADSCs within the microspheres underwent 3D self‐assembly, similar to the self‐organization observed in ultra‐low‐attachment U‐shaped 96‐well plates (Figure 4B).

To visualize microsphere architecture, red and green fluorescent nanoparticles were incorporated into the core and shell compartments, respectively. The resulting distribution showed clear boundaries and accurate structural separation (Figure 4C). Particle size analysis indicated a narrow distribution with excellent uniformity (*n* = 100, Figure 4G).

For functional assessment, the conditioned medium derived from ADSC microspheres (Org‐CM) was compared with conventional 2D conditioned medium (2D‐CM). Transwell migration assays (Figure 4D) and scratch wound healing assays (Figure 4h,i) revealed significantly enhanced migratory ability of HFF‐1 cells and HUVECs in the Org‐CM group. Tube formation assays further demonstrated that Org‐CM markedly promoted capillary‐like network formation by HUVECs (Figure 4E). In addition, CCK‐8 assays confirmed that Org‐CM stimulated proliferation in both cell types (Figure 4F).

**Figure 5:**
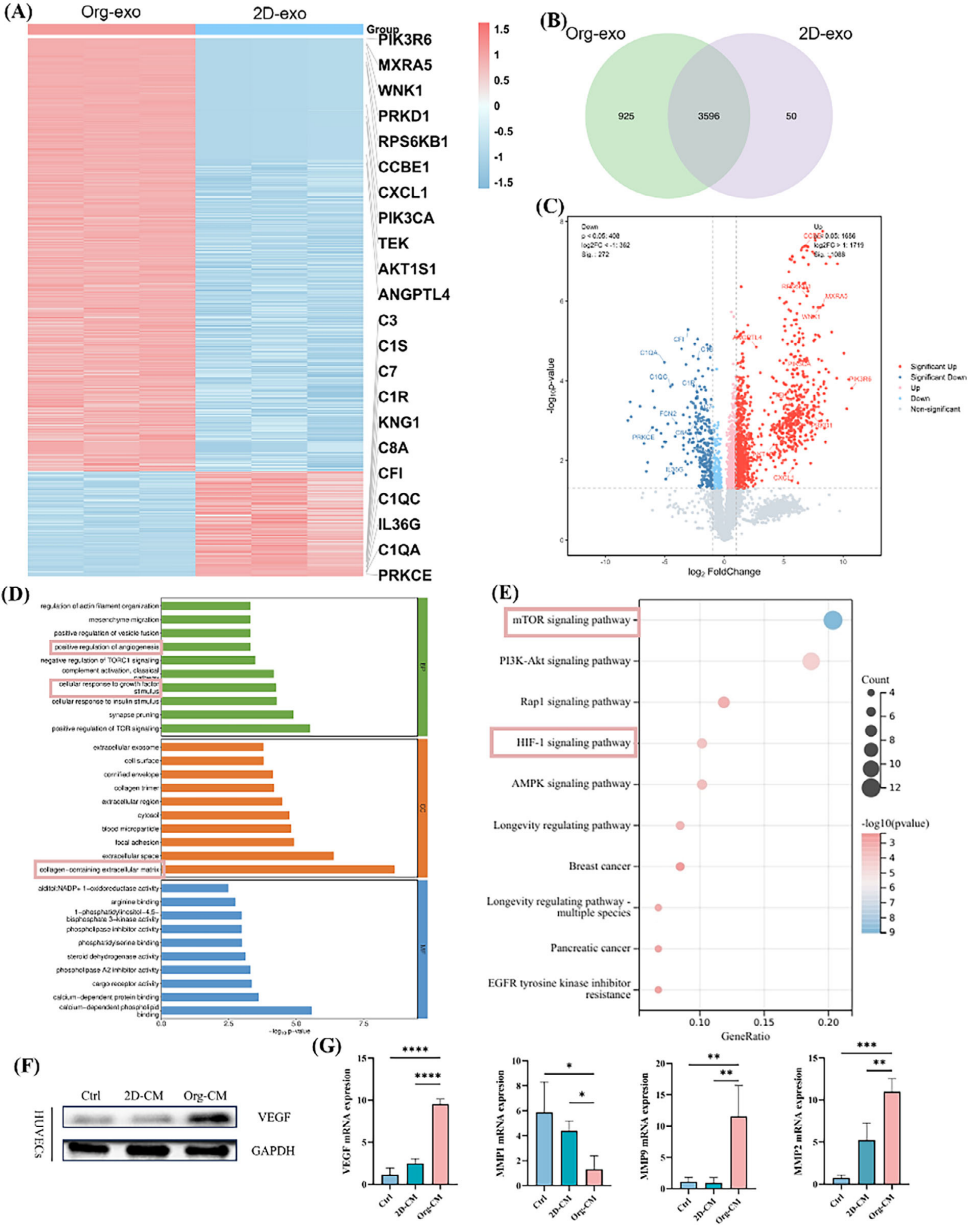
Proteomic analysis of exosomes derived from ADSC microspheres. A) Heatmap of protein expression profiles of exosomes from organoid microspheres (Org‐exo) and 2D cultures (2D‐exo); B) Venn diagram showing shared and unique proteins between the two groups; C) Volcano plot of differential protein expression between Org‐exo and 2D‐exo; D) Gene Ontology (GO) enrichment analysis results, including biological process (BP), cellular component (CC), and molecular function (MF) categories; E) Kyoto Encyclopedia of Genes and Genomes (KEGG) pathway enrichment analysis results; F) Western blot (WB) analysis; G) Quantitative PCR (qPCR) analysis and quantification(*n* = 3, mean ± SD; One‐way ANOVA followed by Tukey's multiple comparisons). Statistical significance is indicated as ^*^ *p* < 0.05, ^**^ *p* < 0.01, ^***^ *p* < 0.001, ns non‐significant.

To further elucidate the molecular mechanisms underlying this paracrine effect, we compared the proteomic profiles of Org‐exo and 2D‐exo (**Figure** **5**), We performed electron microscopy and particle size analysis on exosomes from two sources (Figure S5, Supporting Information). The clustering heatmap (Figure 5A) revealed that Org‐exo exhibited significant upregulation of angiogenesis‐ and matrix remodeling–related molecules such as PIK3CA, TEK,^[^ ^30^, ^31^ ^]^ and CXCL1,^[^ ^32^, ^33^ ^]^ accompanied by downregulation of inflammation‐associated complement components such as C1QA and C1QC. The Venn diagram (Figure 5B) indicated that Org‐exo carried a more diverse protein cargo. The volcano plot (Figure 5C) further demonstrated that the upregulated proteins were primarily enriched in angiogenesis‐ and extracellular matrix remodeling–related factors, which are closely linked to wound repair and neovascularization.

GO analysis (Figure 5D) showed enrichment of differentially expressed proteins in processes associated with angiogenesis, extracellular matrix organization, cell adhesion, and responses to growth factors. KEGG pathway analysis (Figure 5E) highlighted significant involvement of canonical nutrient‐ and angiogenesis‐related pathways, including mTOR,^[^ ^34^, ^35^, ^36^ ^]^ PI3K‐Akt,^[^ ^34^ ^]^ and HIF‐1,^[^ ^37^ ^]^ which were highly consistent with the key molecules identified in the heatmap and volcano plot.^[^ ^34^, ^37^ ^]^ Western blotting and qPCR validation (Figure 5F,G) confirmed these findings, demonstrating significant upregulation of VEGF and MMP2/9^[^ ^32^ ^]^ mRNA levels in HUVECs and increased MMP1 mRNA expression in HFF‐1 cells.

### 2.4 ADSC Microsphere Enhances Cellular Proliferation and Matrix Formation in PV‐SOM

**Figure 6:**
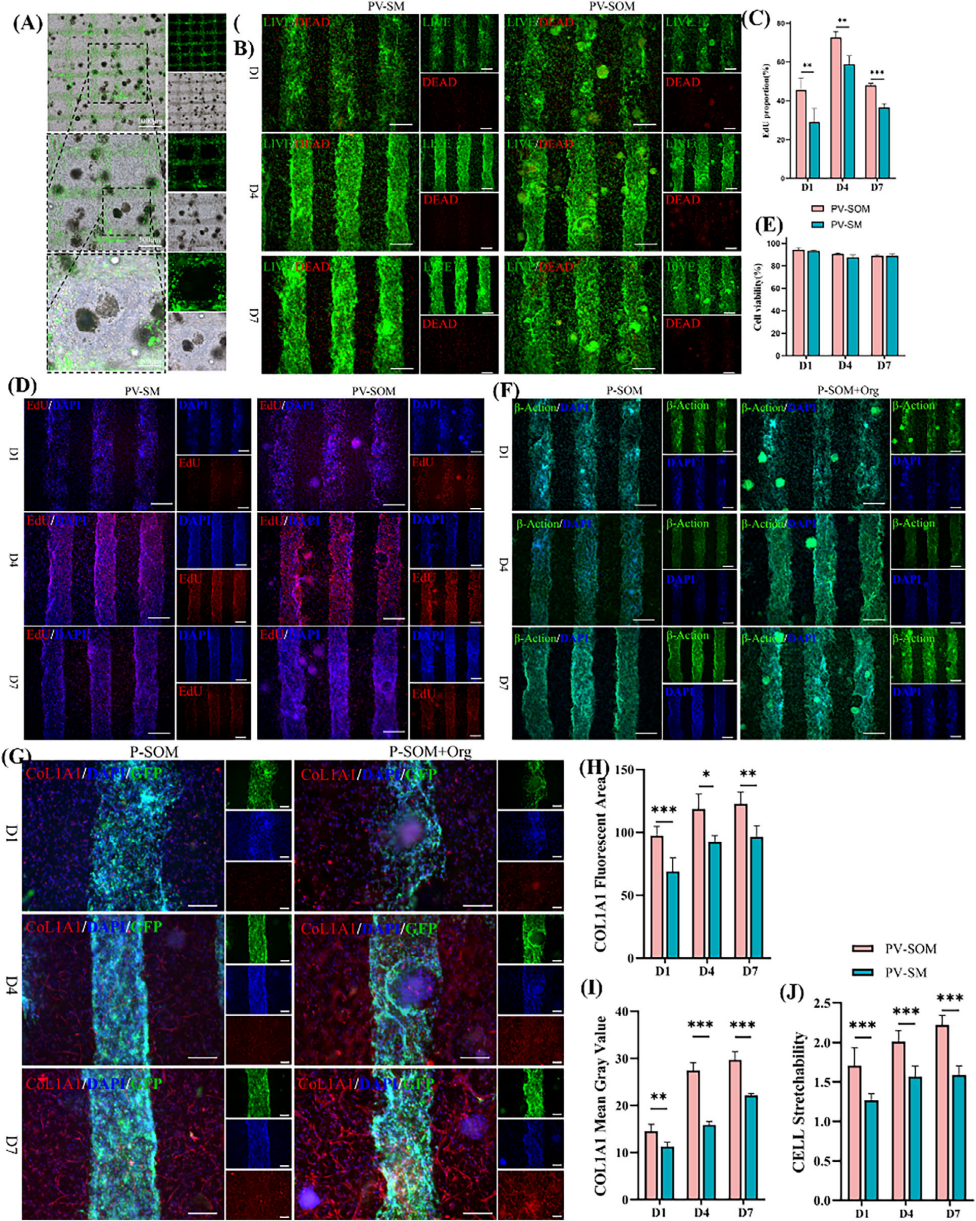
Evaluation of cell activity and matrix production in PV‐SOM containing ADSC microsphere organoids, including A) bright‐field/fluorescence merged images of PV‐SOM showing GFP‐labeled HUVEC vascular structures (green) and scattered ADSC microsphere organoids, B) Live/Dead staining images of PV‐SM and PV‐SOM (scale bar: 200 µm), C) quantitative analysis of EdU‐positive cells, D) EdU staining images (scale bar: 200 µm), E) quantitative analysis of live/dead cell viability, F) β‐actin cytoskeleton staining images (scale bar: 200 µm), G) COLIA1 immunofluorescence staining images (scale bar: 200 µm), H,I) quantitative analysis of COLIA1 fluorescence intensity, and (J) quantitative analysis of cell spreading. *n* = 5 independent samples; significance values were determined by Non‐paired *t*‐test; with statistical significance indicated as ^*^ *p* < 0.05, ^**^ *p* < 0.01, ^***^ *p* < 0.001.

To verify the synergistic effect between pre‐vascularized channels and ADSC microspheres within PV‐SOM, a pre‐vascularized skin model without microspheres (PV‐SM) was used as a control. Bright‐field and fluorescence overlay images revealed that HUVECs‐GFP successfully formed vascular‐like networks within the scaffold, while ADSC microspheres were dispersed between the vascular channels (**Figure** **6**A), indicating that microsphere incorporation did not impair lumen formation. The coaxial 3D printing process of ADSC microspheres, the bio 3D printer, and the microsphere‐containing exaxial bio‐ink are shown in Figure S6 (Supporting Information).

Live/Dead staining showed predominantly green viable cells with few red dead cells in both PV‐SOM and PV‐SM (Figure 6B), and quantitative analysis revealed no significant difference between the two groups (Figure 6E), suggesting overall high viability under high cell‐density conditions.

EdU proliferation assays demonstrated a markedly higher proportion of proliferating cells in PV‐SOM compared with PV‐SM (Figure 6D). Both groups reached a proliferation peak on day 4; however, the positive rate in PV‐SOM approached 80%, significantly higher than in the control. By day 7, PV‐SOM still maintained a proliferation rate above 50% (Figure 6C).

Cytoskeletal staining further indicated enhanced cell spreading in PV‐SOM, with β‐actin fibers clearly organized and tight intercellular connections (Figure 6F). Quantitative analysis confirmed significant improvement in cellular morphology (Figure 6J).

Regarding extracellular matrix production, COL1A1 immunofluorescence staining revealed denser distribution and stronger fluorescence intensity in PV‐SOM (Figure 6G). Quantitative analysis further confirmed significantly upregulated expression (Figure 6H,I), indicating that PV‐SOM accelerates ECM synthesis and maturation.

**Figure 7:**
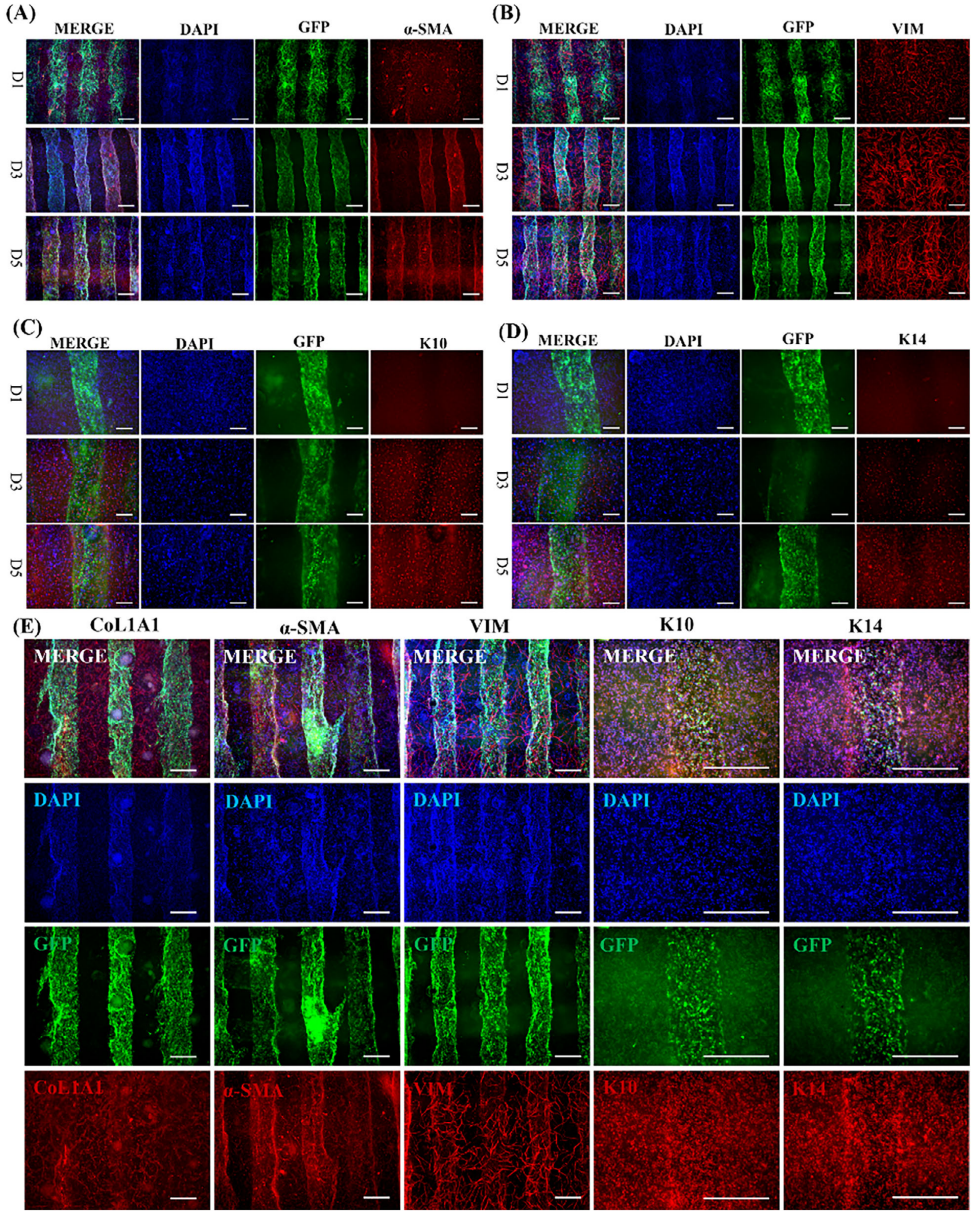
Multilineage characterization of PV‐SOM. A) Immunofluorescence staining of α‐SMA in PV‐SOM constructs at days 1, 3, and 5(scale bar: 500 µm). B) Immunofluorescence staining of Vimentin (VIM) at days 1, 3, and 5 (scale bar: 500 µm). C) Immunofluorescence staining of K10 showing suprabasal keratinocyte distribution at days 1, 3, and 5 (scale bar: 500 µm). D) Immunofluorescence staining of K14 demonstrating basal keratinocyte localization at days 1, 3, and 5 (scale bar: 500 µm). E) Comprehensive multilineage panel of PV‐SOM at day 7, including CoL1A1, α‐SMA, VIM, K10, and K14 (scale bar: 500 µm).

To further assess whether PV‐SOM reconstructs essential epidermal–dermal cellular composition, we performed immunofluorescence staining for four representative skin‐lineage markers. As shown in **Figure** **7**, α‐SMA–positive myofibroblasts (Figure 7A) and Vimentin‐positive fibroblasts (Figure 7B) were widely distributed throughout the hydrogel matrix from day 1 to 5, indicating active mesenchymal remodeling. In the epithelial compartment, K14‐positive basal keratinocytes (Figure 7D) and K10‐positive suprabasal keratinocytes (Figure 7C) were clearly observed, forming early stratified layers adjacent to the vascular structures. By day 5, PV‐SOM displayed a stable epidermal–dermal organization containing multiple native skin lineages (Figure 7E). These findings confirm that PV‐SOM incorporates key cellular populations required for skin architecture and supports its designation as a skin organoid model.

### 2.5 PV‐SOM Enhances Skin Regeneration and Vascularization In Vivo

**Figure 8:**
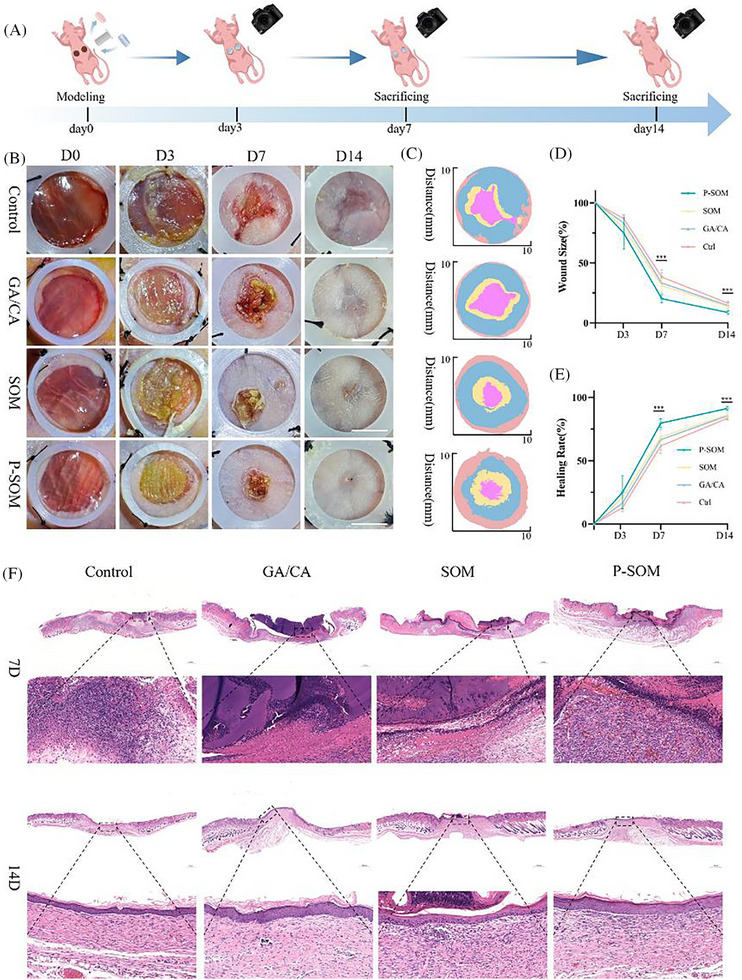
In vivo evaluation of wound healing with pre‐vascularized skin organoids (PV‐SOM), including A) schematic illustration of the full‐thickness skin defect model and treatment procedure, B) gross images of wounds from each group at postoperative days 1, 3, 7, and 14 (scale bar: 1 cm), C) schematic diagram of wound healing, D,E) statistical analysis of wound closure rate (*n* = 6, mean ± SD; One‐way ANOVA followed by Tukey's multiple comparisons), and F) hematoxylin and eosin (H&E) staining images of wound tissues at days 7 and 14 (scale bar: 500 µm). With statistical significance indicated as ^*^ *p* < 0.05, ^**^ *p* < 0.01, ^***^ *p* < 0.001.

To evaluate the in vivo regenerative function of PV‐SOM in large‐area skin defect repair, a full‐thickness dorsal skin defect model was established in mice with four treatment groups: PBS‐treated control (Ctrl), blank GelMA–Col1MA hydrogel (GE/CO), skin model without vascular channels and microspheres (SM), and PV‐SOM containing both HUVEC‐based pre‐vascular structures and ADSC microspheres (**Figure** **8**A).

Wound images were captured on days 0, 3, 7, and 14 post‐surgery. PV‐SOM markedly accelerated wound closure, with significantly smaller wound areas at days 3 and 7 compared with SM, GE/CO, and Ctrl groups (Figure 8B,C). Quantitative analysis of wound closure confirmed this trend, showing that PV‐SOM achieved significantly higher closure rates on day 7, and the difference remained evident at day 14 (Figure 8D,E).

Hematoxylin–eosin (HE) staining revealed distinct differences in tissue repair quality across groups (Figure 8F). On day 7, Ctrl wounds displayed pronounced inflammatory cell infiltration at the wound edge, while the GE/CO group exhibited hydrogel coverage but minimal cellular response. Both SM and PV‐SOM groups showed cell infiltration and epithelialization, with PV‐SOM demonstrating notably richer neovascularization. By day 14, Ctrl wounds remained incompletely repaired, with a thin epidermis and loose dermis. GE/CO and SM groups exhibited partial tissue regeneration but with disorganized architecture. In contrast, PV‐SOM wounds developed a continuous epidermis and well‐organized dermal layer, with parallel collagen fiber alignment and significantly higher vascular density.

### 2.6 PV‐SOM Promotes Vascular Reconstruction and Collagen Remodeling

To assess differences in angiogenesis and repair quality among groups, wound tissues were analyzed by Masson's trichrome staining on days 7 and 14 post‐surgery, followed by type I/III collagen immunohistochemistry and CD31 immunofluorescence on day 14.

**Figure 9:**
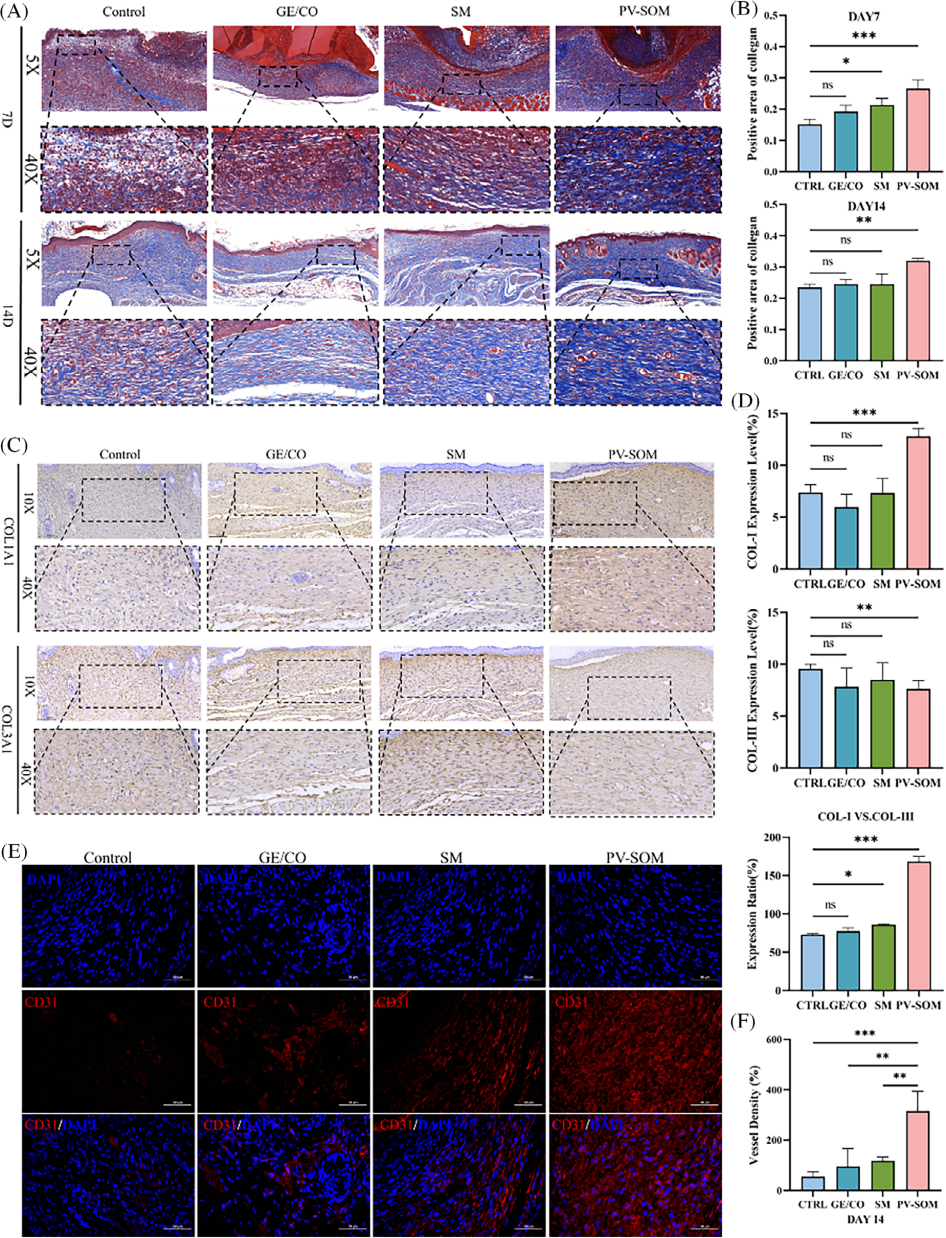
Collagen fiber remodeling and neovascularization in PV‐SOM‐treated wounds, including A) Masson's trichrome staining of wound tissues at days 7 and 14 (scale bar: 100 µm), B) quantitative analysis of Masson's staining (*n* = 6), C) immunohistochemical staining images of type I and type III collagen at day 14 (scale bar: 100 µm), D) quantitative analysis of type I collagen, type III collagen expression, and their ratio (*n* = 6), E) CD31 immunofluorescence staining images at day 14 (scale bar: 50 µm), and F) quantitative analysis of vessel density and vessel area (*n* = 6). *n* = 6 independent samples; significance values were determined by One‐way ANOVA followed by Tukey's multiple comparisons; with statistical significance indicated as ^*^ *p* < 0.05, ^**^ *p* < 0.01, ^***^ *p* < 0.001, ns non‐significant.

Masson's staining (**Figure** **9**A,B) revealed that on day 7, collagen deposition in the dermis was sparse and disorganized in the Ctrl and GE/CO groups, with slight improvement in the SM group. In contrast, the PV‐SOM group exhibited denser collagen fibers with an initial directional alignment. By day 14, the PV‐SOM group showed the most compact and regularly arranged collagen fibers, markedly superior to the other three groups.

Immunohistochemistry for type I and III collagen (Figure 9C,D) demonstrated that on day 14, Ctrl and GE/CO groups were still dominated by type III collagen with relatively low levels of type I collagen. The SM group showed a partial increase in type I collagen, though type III remained predominant. Strikingly, the PV‐SOM group displayed robust upregulation of type I collagen, with the type I/III collagen ratio most closely approximating that of normal skin.

CD31 immunofluorescence (Figure 9E) further confirmed that on day 14, the PV‐SOM group developed abundant CD31⁺ neovessels, with significantly higher vascular density and total vascular area compared with the other three groups. These findings were consistent with the rich neovascularization observed by HE staining in PV‐SOM.

## 3 Discussion

This study aimed to overcome the vascularization bottleneck in organoid research and validated the feasibility of a pre‐vascularized organoid model using skin organoids as a representative application. The PV‐SOM we developed integrates vascular channels and microsphere modules in a single step through coaxial printing combined with microfluidics: the inner phase consists of temperature‐responsive sacrificial materials and HUVECs, while the outer phase contains ADSC microspheres, HFF‐1, HaCaTs, and a highly biomimetic GE/CO hydrogel matrix.

While PSC‐derived skin organoids undergo developmental programs that generate appendage‐forming structures such as follicles and glands, our system does not recapitulate embryonic morphogenesis. Instead, it represents an organoid‐inspired engineered construct that integrates ADSC‐derived microspheres, dermal fibroblasts, and pre‐vascularized channels to reconstruct essential skin architecture and function in vitro. This strategy not only enables efficient construction of functional vascular networks within organoids but also demonstrates the broad applicability of the PV‐XOM concept to vascularization across different organoid systems.

Vascularization is critical for long‐term culture and functional realization of organoids.^[^ ^2^ ^]^ While simple diffusion may suffice for small organoids, it is inadequate for larger ones, especially those exceeding 1 mm in diameter. Without functional vasculature, central regions of large organoids frequently experience hypoxia and nutrient deprivation, leading to necrosis and impaired function.^[^ ^1^, ^2^, ^5^ ^]^ Moreover, vasculature is an indispensable component in organ development and disease pathophysiology.

Our study demonstrated that coaxial printing enables one‐step construction of perfusable vascular channels, with lumen diameters flexibly controlled through printing parameters. With the support of ADSC paracrine activity, endothelial cells rapidly formed closed tubular structures and developed into mature CD31⁺/ZO‐1⁺ vascular networks,^[^ ^38^ ^]^ while ESEM confirmed their biomimetic smooth luminal surfaces. Compared with self‐assembly, co‐culture, or perfusion strategies, coaxial printing offers greater structural designability, single‐step molding, and reproducibility, providing a more efficient approach for constructing functional vascular networks.

ADSC microspheres played a central role in this model by delivering sustained paracrine signals throughout the angiogenesis process, from sprouting to maturation. Microfluidic technology enabled large‐scale, uniform fabrication of core–shell microspheres while preserving ADSC 3D self‐assembly. This structure stabilized the microenvironment, minimized mechanical damage during extrusion, and allowed microspheres to serve as bio‐inks for printing. It also laid the foundation for modular incorporation of different organoid types in PV‐XOM. In vitro experiments demonstrated that ADSC microspheres significantly promoted fibroblast and endothelial cell migration and proliferation, thereby enhancing angiogenesis. Proteomic analyses further revealed that ADSC‐derived exosomes were enriched in pro‐angiogenic and pro‐regenerative molecules acting through pathways such as PI3K–Akt–mTOR,^[^ ^34^, ^35^, ^37^ ^]^ regulating vascularization across multiple stages.

The integration of coaxially printed vascular channels and ADSC microspheres exemplified structural–functional synergy. Sacrificial material–derived hollow lumens supplied nutrients to dense tissues and prevented necrosis, while microspheres continuously released pro‐regenerative and angiogenic factors that promoted the maturation of pre‐formed vascular channels. Staining results demonstrate that PV‐SOM contains major epidermal and dermal cellular populations essential for skin structure. The presence of K14‐positive basal keratinocytes and K10‐positive differentiated layers reflects early epidermal stratification, while Vimentin‐positive fibroblasts and α‐SMA–positive myofibroblasts indicate an actively remodeling dermal‐like matrix. These populations collectively recapitulate the core epidermal–dermal architecture of native skin. Although PV‐SOM does not undergo iPSC‐driven organogenesis or form appendages such as follicles or glands, its adult‐stem‐cell origin, multi‐lineage composition, and biomimetic 3D arrangement justify its designation as a skin organoid model, distinguishing it from both traditional tissue‐engineered skin constructs and true iPSC‐derived skin organoids.

Their complementary effects accelerated vascular closure, guided orderly ECM remodeling in vitro, and translated into robust in vivo outcomes. PV‐SOM significantly promoted wound closure and angiogenesis at early time points and, by day 14, achieved complete epidermis formation, orderly dermal architecture, and a type I/III collagen ratio approaching that of normal skin. These findings confirm that PV‐SOM not only enables rapid construction of perfusable vasculature in vitro but also supports vascular integration and high‐quality skin regeneration after transplantation.

The application of biomimetic composite materials provided the final cornerstone for vascularized organoid construction. The GE/CO hydrogel combined superior mechanical properties with biomimetic ECM characteristics. It exhibited optimal tensile and compressive strength, controllable degradation, and reduced swelling, while offering abundant adhesion sites and a physiologically relevant collagen environment. These properties promoted cell adhesion, migration, and functional activity, establishing GE/CO as a broadly applicable matrix for pre‐vascularized organoid construction.

Nevertheless, several limitations remain. First, in vivo studies were restricted to a murine large‐area skin defect model, and clinical feasibility requires further validation. Second, mechanistic investigations focused mainly on the paracrine effects of ADSC microspheres and angiogenic signaling, without fully elucidating deeper regulatory mechanisms. Finally, the generalizability of the PV‐XOM strategy to more complex organoids such as liver or kidney will require incorporation of multiple cell types and finely tuned microenvironmental cues.

In summary, the successful construction of PV‐SOM validates the PV‐XOM pre‐vascularization strategy as an effective solution to the vascularization bottleneck in organoid systems. Applied here to large‐area skin defects, PV‐SOM achieved high‐quality wound repair and neovascularization. This approach—combining coaxial printing for one‐step fabrication of perfusable vascular channels with microfluidic generation of organoid microspheres embedded in a biomimetic GE/CO matrix—offers a versatile and broadly applicable strategy for the creation of pre‐vascularized organoids. These findings provide a new paradigm for regenerating complex tissues and pave the way for vascularization of diverse organoid systems.

## 4 Experimental Section

### Experimental Design

This study aimed to construct and validate a modular prevascularized skin organoid model (PV‐SOM). The overall experimental workflow included: i) culture and expansion of human‐derived cells; ii) preparation and characterization of a biomimetic GelMA–Col1MA (GE/CO) composite hydrogel; iii) one‐step fabrication of PV‐SOM using coaxial 3D printing and microfluidic technology; and iv) in vitro functional assays and in vivo validation in a large‐area skin defect model.

### Cell Culture and Experimental Animals’ Welfare

Human adipose‐derived mesenchymal stem cells (ADSCs, Cat. No. ZQY007, Zhongqiao Xinzhu, China) (RRID: CVCL_WG56). ADSCs were expanded in the corresponding culture medium (Cat. No. ZMY007, Zhongqiao Xinzhu). Cells were maintained at 37 °C in a humidified 5% CO_2_ incubator, and culture medium was refreshed every 2–3 days depending on cell density. ADSCs between passages 3–6 were used for the fabrication of ADSC microspheres and subsequent PV‐SOM construction.

Human umbilical vein endothelial cells (HUVECs)(RRID: CVCL_9Q53), GFP‐labeled HUVECs (Lonza, USA), human Immortalized Keratinocytes(HaCaTs, Cat. No. iCell‐h066, ICell Bioscience Inc., Shanghai) (RRID: CVCL_0038), and human foreskin fibroblasts (HFF‐1, Shanghai Shuocheng Biotechnology Co., Ltd.) (RRID:CVCL_3285). Cells were cultured in DMEM (C11995500BT, Gibco, Guangzhou, China) containing 10% FBS (164210, Procell, China) and 1% penicillin–streptomycin (15140122, Gibco) under standard conditions. Cells were used at passages 4–8 for printing.

Immunodeficient male BALB/c mice (6–8 weeks, 20–25 g) were purchased from Zhuhai BesTest Biotechnology Co., Ltd. Mice were housed under SPF conditions and acclimated for 1 week before surgery. All animal procedures were approved by the Animal Ethics Committee of the Third Affiliated Hospital of Sun Yat‐sen University (RW‐IACUC‐25‐0058) and complied with relevant guidelines.

### Hydrogel Preparation

The hydrogel for the PV‐SOM shell consisted of gelatin methacryloyl (GelMA, EFL‐GM‐60, EFL, Suzhou, China), collagen type I methacrylate (Col1MA, BEO‐Col1MA‐001, BeoGene, Guangzhou, China), and photoinitiator LAP (EFL‐LAP, EFL). GelMA (10% w/v) and Col1MA (3% w/v) were dissolved in DMEM containing 0.25% LAP under magnetic stirring at 37 °C overnight in the dark, then mixed at a 1:1 volume ratio to obtain the GE/CO precursor solution. The core gelatin phase (G108398, Aladdin, China) was prepared by dissolving 5% (w/v) gelatin in DMEM at 37 °C. All solutions were sterilized using 0.22 µm filters and stored at 37 °C until use.

### Hydrogel Characterization

Mechanical Testing and Rheology: GE/CO and GelMA precursor solutions were cross‐linked into cubic hydrogels (5 mm side length). Tensile strength, elongation at break, and compressive resistance were measured using a universal testing machine (HDW‐20, China). Rheological properties were assessed with a rotational rheometer (MCR92, Anton Paar, Austria), including shear‐thinning behavior (0.1–100 s^−1^) and frequency sweep (0.1–10 Hz), to obtain storage modulus (*G*′) and loss modulus (*G*″).

Enzymatic Degradation: Hydrogels (5 mm cubes) were incubated in collagenase I solution (1 mg mL^−1^) at 37 °C, weighed every 2 h until complete degradation.

Swelling: Lyophilized hydrogels were weighed (W_0_), incubated in PBS (37 °C), and reweighed at 1, 4, 8, and 24 h (Wt). Swelling ratio was calculated as SR = (Wt − W_0_)/W_0_ × 100%.

Microstructure: Freeze‐dried samples were sputter‐coated with gold and imaged by field‐emission scanning electron microscopy (Sigma 300, Zeiss, Germany) to assess pore structure and fiber distribution.

Photo‐crosslinking: Gelation time of GE/CO was measured under 405 nm blue light (100 mW/ m^−^^2^).

Printability: Line‐printing tests were performed using a bioprinter to evaluate extrusion continuity and shape fidelity.

Cytocompatibility: HUVECs and HFF‐1 (1 × 10⁶ cells mL^−1^) were encapsulated in GelMA or GE/CO hydrogels and subjected to Calcein‐AM/PI staining on days 1 and 7. Additionally, cubic hydrogels (5 mm) were incubated in DMEM (1 cm^2^ mL^−1^, 37 °C, 24 h) to prepare extracts for CCK‐8 proliferation assays.

### PV‐SOM Fabrication

ADSCs were digested into single‐cell suspensions in gelatin core solution (3 × 10⁷ cells mL^−1^) to generate ADSC microspheres using a droplet‐based organoid printer (XE‐Q10, Qingyuan Zhixin, Shenzhen, China). Oil flow rate was 35 mL h^−1^, with inner/outer aqueous phases at 10 µL min^−1^. After printing, microspheres were centrifuged (600 rpm, 2 min) to remove oil and stored at 37 °C. HUVECs were suspended in gelatin core solution (3 × 10⁷ cells mL^−1^), HaCaT cells were mixed with HFF‐1 fibroblasts in the GE/CO hydrogel prior to printing at a density of 1 × 10⁶ cells mL^−1^. Coaxial extrusion bioprinting was performed (Medprin, Guangzhou, China) with a nozzle (inner 22G, outer 17G, Nayi Instruments, Changsha, China). Core: HUVEC–gelatin solution; shell: GE/CO–HFF‐1 with ADSC microspheres. Parameters: core 60 µL min^−1^, shell 400 µL min^−1^, platform 50 mm min^−1^, printing temperature 23 °C, stage 10 °C, scaffold size 13 × 13 × 1 mm. After printing, constructs were crosslinked under 405 nm blue light (100 mW cm^−^^2^, 20 s), rinsed with PBS, and cultured in complete medium at 37 °C. The gelatin core dissolved at 37 °C, forming hollow vascular channels.

To ensure reproducibility, all GelMA and Col1MA hydrogels were prepared from the same production batch, aliquoted, and stored under identical conditions. Printing parameters (flow rate, nozzle gauge, temperature, and platform speed) were standardized and verified through calibration prints prior to each session to reduce batch‐to‐batch and day‐to‐day variability.

### Exosome Extraction and Conditioned Medium Preparation

To prepare ADSC microsphere‐conditioned medium (Organoid‐CM), supernatants from 2D ADSC culture (P6) and microsphere culture were collected. Sequential centrifugation was performed: 300 × g (10 min) to remove live cells, 2000 × g (30 min) for dead cells, and 10 000 × g (30 min) for debris. The supernatant was filtered (0.22 µm) and ultracentrifuged at 100 000 × g (70 min, 4 °C, Optima MAX‐XP, Beckman Coulter, USA). Pellets were washed with PBS and recentrifuged, resuspended to 1 × 10^1^⁰ particles mL^−1^, then diluted to 1 × 10⁹ particles mL^−1^ in complete medium.

### In Vitro Functional Assays

Scratch Assay: HFF‐1 and HUVECs were seeded in six‐well plates to 80% confluence, serum‐starved overnight, scratched with 200 µL tips, rinsed with PBS, and cultured in either conditioned medium or serum‐free DMEM. Images were taken at 0, 12, and 24 h and quantified with ImageJ.

Transwell Migration: HUVECs/HFF‐1 (1 × 10⁵ cells mL^−1^, 200 µL) were seeded in the upper chamber; lower chambers contained 600 µL conditioned or complete medium. After 24 h (37 °C), migrated cells were fixed, stained with crystal violet, imaged, and quantified.

qRT‐PCR: After 48 h of treatment, total RNA was extracted, reverse transcribed, and amplified using the SYBR Green method. Briefly, Evo M‐MLV reverse transcription reagent premix (Cat. No. AG11706, ACCURATE BIOTECHNOLOGY (HUNAN) CO., LTD, Changsha, China) was used according to the manufacturer's instructions, with GAPDH as the internal control and the 2^−^ΔΔCt method used.

Western Blot: After 48 h treatment, proteins were extracted using RIPA buffer, quantified (BCA), resolved by SDS‐PAGE, transferred to PVDF membranes, blocked, incubated with VEGF and GAPDH antibodies (1:1000, 4 °C, overnight), HRP‐secondary (1:5000, 1 h), and visualized with ECL. Experiments were repeated in triplicate.

### Proteomics

Exosomes were isolated from supernatants of 2D ADSC and ADSC microsphere cultures via ultracentrifugation as above. Samples were submitted to OE Biotech Co., Ltd. (Shanghai, China) for proteomic sequencing and analysis.

### In Vivo Animal Experiments

Male BALB/c mice (6–8 weeks, 20.0 ± 5.0 g, *n* = 24) were used for large‐area skin defect modeling. Under isoflurane anesthesia, full‐thickness circular wounds (10 mm diameter) were excised from the dorsal skin. Mice were randomly assigned (*n* = 6/group): i) Ctrl (PBS), ii) GE/CO (acellular hydrogel), iii) SM (three‐cell mixture without vascular channel and microspheres), and iv) PV‐SOM (complete model). Anti‐contraction rings were applied to prevent wound contraction. Wound closure was photographed on days 0, 3, 7, and 14, quantified by ImageJ. All mice received standard chow throughout. On day 14, mice were euthanized with CO_2_, and wound tissues were collected, fixed in 4% paraformaldehyde, dehydrated, paraffin‐embedded, and sectioned for histology.

### Histology and Wound Sections

Hematoxylin–eosin (H&E) and Masson's trichrome staining were performed. For H&E, rehydrated samples were stained with hematoxylin (2 min), eosin (6 min), dehydrated, and mounted. For Masson's staining, sections were processed sequentially with Weigert's iron hematoxylin, Biebrich scarlet‐acid fuchsin, and aniline blue, then mounted.

### Immunofluorescence (IF) and Immunohistochemistry (IHC)

For IF, samples were fixed, permeabilized (0.2% Triton X‐100), blocked, and incubated overnight at 4 °C with anti‐COL1A1, anti‐CD31, and anti‐ZO‐1 (1:500). Fluorescent secondary antibodies (1:500, 1 h, RT) were applied, followed by imaging with a high‐content fluorescence microscope. For epidermal and dermal lineage characterization, samples were incubated with primary antibodies against K10 (1:300), K14 (1:300), Vimentin (1:500), and α‐SMA (1:500) overnight at 4 °C, Fluorescent secondary antibodies (1:500, 1 h, RT) were applied, followed by imaging with a high‐content fluorescence microscope.

For IHC, antigen retrieval (sodium citrate, 15 min) was performed, sections were blocked (3% BSA, 30 min), incubated with primary antibodies (anti‐Collagen I/III, 4 °C, overnight), HRP‐secondary (1 h), developed with DAB, counterstained with hematoxylin, and imaged.

### Statistical Analysis

All quantitative analyses were performed using unprocessed raw data, and results are presented as mean ± standard deviation (SD). The sample size (n) for each experiment was specified in the corresponding figure legends. Statistical analyses were conducted using GraphPad Prism 9.5. For comparisons between two independent groups, statistical significance was assessed using a two‐tailed non‐paired Student's *t*‐test. For experiments involving more than two groups, one‐way ANOVA followed by Tukey's multiple comparisons test was applied. Statistical significance was defined as *p* < 0.05. Exact statistical tests, n values, and adjusted p values are reported in the figure legends, and the following notation was used throughout the manuscript: ^*^ *p* < 0.05, ^**^ *p* < 0.01, ^***^ *p* < 0.001, with “ns” indicating non‐significance.

## Conflict of Interest

The authors declare no conflict of interest.

## Supporting information



Supporting Information

## Data Availability

The data that support the findings of this study are available in the supplementary material of this article.
