# Supplementary material for: One‐Step Coaxial 3D Printing of Pre‐Vascularized Skin Organoid Models with ADSC Microspheres for Enhanced Wound Healing
Source: Adv Sci (Weinh). 2025 Nov 29;13(9):e17409. doi: 10.1002/advs.202517409 (PMC12904007; doi:10.1002/advs.202517409)
Supplement: Supplementary file 1 — Supporting Information [file ADVS-13-e17409-s001.docx]

**Supporting Information**


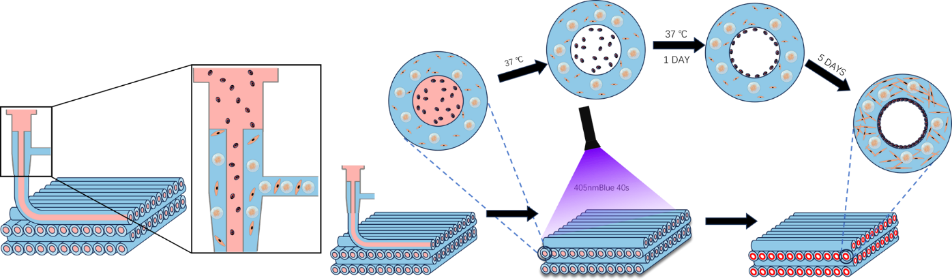


**Figure S1. Schematic diagram of coaxial 3D printing**


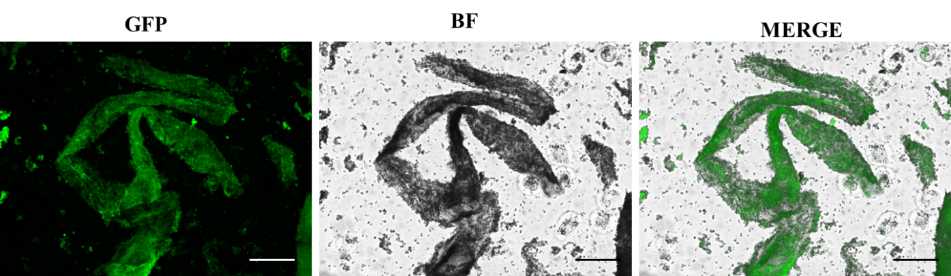


**Figure S2.** **After the seventh day of in vitro culture, the vascular structure was observed under a microscope after dissolving the GelMA (scale bar: 200 μm).**


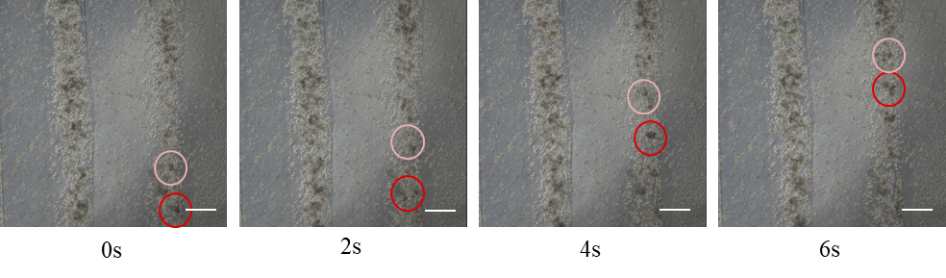


**Figure S3. Cells flow within pre-vascular channels.** From 0 seconds to 6 seconds, the cells within the red and pink frames move over 500 micrometers (scale bar: 200 μm).


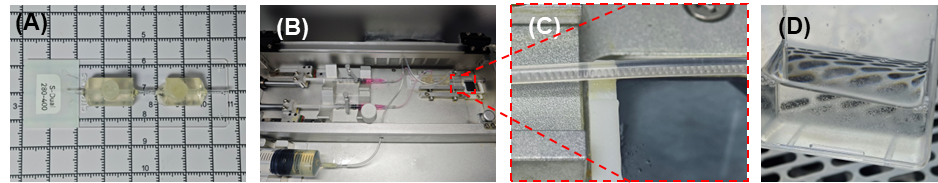


**Figure S4. Microfluidic Printing.** (A) Core-shell dual-channel printing chip, (B) organoid printer, (C) non-photopolymerized microsphere organoids; (D) ADSC organoid microspheres in culture for exosome extraction.


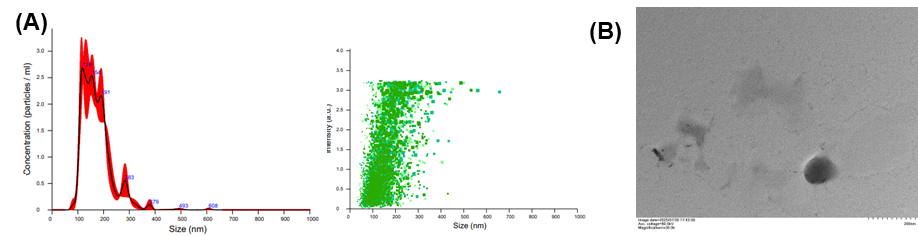


**Figure S5. Characterization of Organoid Exosomes.** (A) Analysis of exosome particle size and concentration, (B) Electron microscopy images of exosomes.


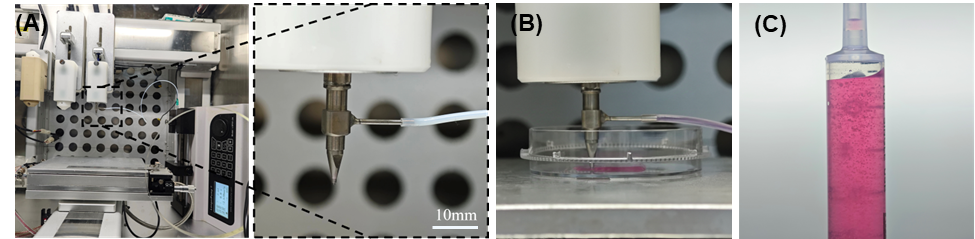


**Figure S6. The coaxial printing process.** (A) Extrusion-based 3D printer and print head, (B) PV-SOM in the process of being printed, (C) Highly biomimetic bio-ink in the outer axis containing ADSC microspheres.
